# Prevalence of physical activity and dietary patterns as risk factors for cardiovascular diseases among semi-urban dwellers in Ibadan, Nigeria

**DOI:** 10.4314/ahs.v22i3.36

**Published:** 2022-09

**Authors:** Posi Emmanuel Aduroja, Yetunde Olufisayo John-Akinola, Mojisola Morenike Oluwasanu, Oladimeji Oladepo

**Affiliations:** Department of Health Promotion and Education, Faculty of Public Health, College of Medicine, University of Ibadan, Nigeria

**Keywords:** Physical activity, diet, semi-urban, community

## Abstract

**Background:**

Physical inactivity and unhealthy diet are leading risk factors for cardiovascular diseases globally. Limited studies have assessed the prevalence of these risk factors in community-based settings in Nigeria.

**Objectives:**

This study assessed the prevalence of physical activity and the dietary pattern of residents in selected semi-urban communities in Ibadan, Nigeria.

**Methods:**

This was a cross-sectional study carried out among 500 randomly selected residents from two semi-urban communities. Multi-stage random sampling technique was used to select households and participants. Data were collected using a pretested modified version of the WHO STEPS instrument. Descriptive and inferential statistical analyses were determined at 5% level of significance.

**Results:**

The mean age was 35.36 ± 12.24 and a mean household size of 4.07 ± 1.85. Majority (87.2%) of the respondents engaged in low physical activity (< 150–300 min/wk). Consumption of fruits and vegetables was low among respondents at 33% and 36.4% respectively. The employment status of respondents was significantly related to expected workplace physical activity level (χ2=11.27; P=0.024).

**Conclusions:**

This study highlights the need for the development and implementation of community-driven, multi-layered public health promotion initiatives across different settings.

## Background

The well-being of individuals is a function of their level of physical activity^1^–^4^. Physical activity has a positive influence, not only on the physical fitness of individuals and reduction in the risk of cardiovascular diseases (CVDs)^5^,^6^ but also on improving the cognitive and psychological well-being of individuals^7^,^8^. Reports from World Health Organization^9^ showed that low physical activity-related Non-communicable Diseases (NCDs) contributed up to 3 million deaths in sub-Saharan Africa (SSA). From projections in literature^10^, this is expected to have increased to about 80% in the year 2020. Globally, low physical activity has been found to contribute to 6% of coronary heart disease burden, 7% of type-2 diabetes disease burden, 10% of breast cancer disease burden and 10% of colon cancer disease burden^11^.

Low physical activity is responsible for 9% of the global mortality rate and it is highlighted as the second leading cause of death in the US and fourth globally^11^. Data collected across one hundred and twenty-two (122) countries by Hallal and colleagues^20^ showed that 31% of adults worldwide are inactive, with a high likelihood of an increase in subsequent years.

Low physical activity is fast becoming an epidemic in West African countries, and it is higher in this region compared to other middle-income countries^12^. In Nigeria, several reports have shown low physical activity levels between 25% to 32%^12^,^21^–^23^, with NCDs accounting for around 25% and 35% of all deaths in Nigerian men and women, respectively^24^.

Studies carried out in Nigeria^16^ and Cameroon^17^ reported a sedentary lifestyle in workplaces, especially in urban areas; this has been attributed to low physical activity as a result of increase in white-collar jobs and long hours of sitting^12^,^18^. Assah and colleagues^19^ and Ejechi^18^, however reported that physical activity is higher in traditional rural settings of Africa as a result of farming, hunting, and fishing.

It has been documented that obesity and sedentary lifestyle coexist and are both associated with CVDs^25^ and this calls for public health urgent attention. Furthermore, putting into perspective that Nigeria is the sixth-largest and Africa's most populated country, between 48.9 and 62.6 million Nigerians are at risk of NCDs. This number is more than the entire population of South Africa, Kenya and Uganda. With an estimated increase in life expectancy by 0.68 years^11^, the need for the elimination of low physical activity within the Nigerian population is most vital. Studies across the world have shown that while individuals may contemplate engaging in physical activity, the environment they live in has a strong influence on their readiness to do so^26^–^30^.

Poor dietary pattern in combination with low physical activity increases the risk for the development of CVDs. In recent years, there has been a rapid increase in the number of fast foods restaurants, commercial food canteens, food hawkers, and street vendors, which are patronised by people of different ages^13^. According to Abubakari^12^, urbanisation has led to the increased consumption of food items high in fat and sugar with high levels of calories. In combination with low physical activity and sedentary lifestyle reported, this further puts a substantial number of the Nigerian population at risk of CVDs. Bamiro^13^ stated that urban residents purchase 37.9% of the food they consume; these foods are referred to as food-away-from-home (FAFH). The consumption of FAFH has increased across the globe, both in developed and developing countries. Food-away-from-home has been established to have lesser nutritious content and high caloric content compared to food prepared at home^14^. Also, eating away from home has been linked to overweight and obesity in adults and children, especially abdominal obesity^13^. This constitutes a major risk factor to the increasing prevalence of CVDs^15^.

In perspective, published studies by Sun et al.^31^ and Almeida et al.^32^, and a systematic review conducted by Daskalopoulou et al.^33^ reported a positive association between physical activity and healthy aging. Weight gain is correlated with both physical activity and dietary pattern^34^,^35^. Diet and physical activity are considered to be major components of a healthy lifestyle and cannot be separated. Limited studies have assessed the prevalence of low physical activity and dietary pattern in community-based settings in Nigeria. Thus, this study's objectives are as follows:

i. To identify the consumption pattern of fruits and vegetables among respondents.

ii. To identify the consumption pattern of sugar-sweetened drinks, pastries and food-away-from-home among respondents.

iii. To determine the level of physical activity among respondents

## Methods

### Study design and setting

This was a cross-sectional study nested into a larger study which documented bio-behavioural data on NCD risk factors using the ODK tool. A community-based study was carried out among inhabitants of two semi-urban communities in Ibadan North Local Government Area, Oyo State, Nigeria from fourteenth November to third December 2018. The LGA has an estimated population of 856,988 and sanitary conditions are poor as the majority of houses do not have access to potable water and toilets.

### Participant size and sampling technique

The sample size for the study was determined using the Leslie Kish formula, a proportion of physical activity of 32% from a previous study report^23^, 95% confidence level, 5% margin of error, and a design effect of 1.5. The final sample size was determined to be 334 and increased to 500 respondents (approximately 50%) to cater for incomplete responses and to increase the number of respondents to cover a larger portion of the communities. The participants consisted of 500 community members aged 18–65 years who were selected for the study from the total community population using a two-step multistage sampling of households and residents. For the first stage, a systematic random sampling technique was used to select every third households in the selected community, however, household who did not consent were not enrolled and the subsequent household was enrolled upon giving consent. The second stage, from each of the selected households, one participant was selected by the ballot method.

### Variables and definitions

Variables measured in this study are respondents' physical activity measured on a scale of World Health Organisation (WHO) recommendation of 150 minutes per week as moderate-intensity physical activity and 300 minutes per week as vigorous-intensity physical activity, consumption of fruits, vegetable, pastries, sugar-sweetened drinks, and FAFH was measured as regular (4 or more days of consumption in a week) and non-regular (less than 4 days of consumption in a week). These variables were self-reported by respondents.

### Data collection instrument and measurement

Data was collected using the modified version of the WHO STEPS instrument; the tool has two major parts: socio-demographic characteristics and behavioural profile. The modified instrument was translated into the local language Yoruba language, which was further back-translated into English language to ensure items retained their original meaning. Data was gathered through an electronic data capture tool (ODK Collect) which was interviewer-administered. Research assistants and supervisors were trained for two days, the training included research ethics, data collection procedures, and contents of the instrument to increase the quality of the data. The data collection procedure and tools were pretested in urban communities that shared similar characteristics to the study locations. Supportive supervision was carried out by supervisors daily during the data collection period in the two selected communities. The blood pressure reading was taken after the interviewer-administration of questionnaires using an electronic sphygmomanometer; weight and height were measured using a weighing scale and a stadiometer, respectively.

The collected data was exported into the IBM Statistical Package for Social Sciences version 21.0 for analysis. Descriptive and inferential statistical analyses were carried out. Chi-square test was used in testing for a significant relationship between respondents' demographic characteristics and dietary patterns variables and also physical activity, Analysis of Variance (ANOVA) was used to test for significant difference and Duncan Multiple Range Test (DMRT) was used in ranking variation in mean scores of fruits and vegetable consumption.

## Results

### Socio-demographic characteristics

There were 500 respondents in the study. The mean age of respondents was 35.36 ± 12.24, and more than half (54.2%) were between the age of 26 and 45 years. The majority (70.6%) of the respondents were females, over half (52.6%) had completed secondary education, and the majority (88.8%) were of Yoruba ethnicity. Many (62.4%) were married and slightly more than half (51%) were Christians. More than two-thirds of the respondents (65.8%) had lived in the study location for 10 years or less, 72.6% are self-employed, 67.2% earn a regular monthly income of 20,000 naira ($51) or less which is below the minimum wage allocation in Nigeria as at the time this study was conducted. Household size ranged from 1 to 10 individuals with a median of 4 (Table 1).

**Table 1:** General demographic characteristics of the study sample (N = 500)

Variables	Frequency	Percentage
Sex		
Male	147	29.4
Female	353	70.6
Age Group*		
Youth (18 – 25 years)	119	23.8
Adult Middle age (26 – 45 years)	271	54.2
Adult Mature age (46 – 65 years)	110	22.0
Education		
No formal schooling	49	9.8
Primary education	78	15.6
Secondary education	263	52.6
College/University education	110	22.0
Ethnic group		
Yoruba	444	88.8
Igbo	26	5.2
Hausa	4	0.8
Others (Akwa Ibom, Benue, Cross River, Delta, Edo, Ibibio, Kogi, Tapa & Non-Nigerian)	26	5.2
Marital status		
Never Married	142	28.4
Currently married	320	64.0
Not married	38	7.6
Religion		
Christianity	255	51.0
Islam	245	49.0
Years of residence**		
10 years or less	329	65.8
11 years to 20 years	75	15.0
21 years to 30 years	60	12.0
More than 30 years	36	7.2
Employment status		
Employed	64	12.8
Self-employed	363	72.6
Unemployed	73	14.6
Monthly income***		
No income	34	6.8
20,000 naira or less	336	67.2
More than 20,000 naira	130	26.0
Household size****		
1 member	41	8.2
2 to 4 members	274	54.8
5 or more members	185	37.0

* Mean age = 35.36 ± 12.24

** Mean years of residence = 11.41 ± 11.64

*** Mean monthly income = 19714.60 ± 22975.14

**** Median household size = 4

### Consumption of fruits and vegetables

Regular consumption of fruits was low among respondents (33%), 73.9% of respondents who consumed fruits regularly were female. Married respondents (64.2%), respondents who had lived for 10 years or less within the study site (61.2%), self-employed (72.7%), respondents who earn 20,000 naira or less (67.9%) and respondents from a household with 2 to 4 members (59.4%) consumed fruits more regularly, years of residency was found to be significantly related to regular consumption of fruits X2 (3, N = 500) = 12.80 (Table 2).

**Table 2:** Dietary pattern of the sample according to their demographic characteristics

Variables	Regular consumption of fruits *n* = 165 (33.0%) %	Regular consumption of vegetables *n* = 182 (36.4%) %	Regular consumption of sugar-sweetened drinks *n* = 121 (24.2%) %	Regular consumption of pastries *n* = 88 (17.6%) %	Regular consumption of FAFH *n* = 38 (7.6%) %
Sex					
Male	26.1	27.5	28.9	28.4	78.9*
Female	73.9	72.5	71.1	71.6	21.1*
Age Group					
Youth (18 – 25	23.0	17.0*	33.9*	37.5*	36.8
years)	49.1	52.2*	53.7*	48.9*	47.4
Adult (26 – 45 years)	27.9	30.8*	12.4*	13.6*	15.8
Middle age (46 – 65 years)					
Marital status					
Never Married	30.9	27.5	24.0	21.6	18.4
Currently married	64.2	65.4	66.1	68.2	71.1
Not married	4.8	7.1	9.9	10.2	10.5
Years of residence:					
10 years or less	61.2*	55.5*	66.9	65.9	68.4
11 years to 20 years	11.5*	14.8*	14.9	15.9	15.8
21 years to 30 years	18.8*	19.2*	13.2	15.9	10.5
More than 30 years	8.5*	10.4*	5.0	2.3	5.3
Employment status:					
Employed	15.2	12.1	12.4	10.2	10.5
Self-employed	72.7	73.6	74.4	75.0	86.8
Unemployed	12.1	14.3	13.2	14.8	2.6
Monthly income:					
No income	8.5	8.8	7.4	9.1	5.3
20,000 naira or less	67.9	61.0	62.0	69.3	55.3
More than 20,000	23.6	30.2	30.6	21.6	39.5
Household size:					
1 member	8.5	8.2	5.0	9.1	13.2
2 to 4 members	59.4	54.4	57.9	46.6	60.5
5 or more members	32.1	37.4	37.2	44.3	26.3

* *P*< 0.05

Regular consumption of vegetables was low among respondents (36.4%). Statistically, there was a significant relationship was found between regular consumption of vegetable and respondents age X2 (2, N = 500) = 15.72 with the adult respondents significantly consuming more than any other age groups, and also with respondents' years of residence X2 (3, N = 500) = 21.26 with respondents with 10 or fewer years of residency consuming more vegetables (Table 2).

### Consumption of sugar-sweetened drinks, pastries and food-away-from-home

Respondents' consumption of Sugar-Sweetened Drinks (SSD) was low at 24.2%, while female respondents were found to be higher consumers of SSD (71.1%), significantly, Respondents' age X2 (2, N = 500) = 13.52 was related to SSD consumption, more among adult respondents (Table 2)

Pastries consumption was low among respondents (17.6%). While this was significantly related to respondents' age X2 (2, N = 500) = 12.38, the adults were found to consume more, likewise significantly related to pastries consumption was employment status X2 (4, N = 500) = 20.11 with self-employed respondents consuming more (Table 2).

Regular eating of food not cooked at home was very low among respondents (7.6%), this is however more pronounced among the male respondents (78.9%), adult (47.4%), married respondents (71.1%), residents of 10 years or less (68.4%), self-employed (86.6%), earners of 20,000 naira or less (55.3%) and household of 2 to 4 members (60.5%). Respondents' age X2 (1, N = 500) = 48.64 was significantly related to FAFH consumption (Table 2).

### Prevalence of physical activities

The prevalence of physical activities across respondents' demographic characteristics showed that vigorous-intensity physical activity was very low (15.6%) among respondents. Higher vigorous-intensity activities were reported by females (59%), adults (64.1%), and self-employed (74.4%) respondents. A statistically significant relationship was found between vigorous-intensity physical activity and respondents' sex X2 (1, N = 500) = 6.02 (Table 3). Moderate-intensity physical activity was equally very low (20.8%) among respondents, this was however more pronounced among female respondents (61.5%), adult (54.8%), and self-employed (72.1%) respondents. A statistically significant relationship was found between moderate-intensity physical activity and respondents' sex X2 (1, N = 500) = 5.20.

**Table 3:** Physical activity of sample according to demographic characteristics

Variables	vigorous-intensity physical activity		moderate-intensity physical activity	
	
	Yes *n* = 78 (15.6%) %	No *n* = 422 (84.4%) %	Yes *n* = 104 (20.8%) %	No *n* = 396 (79.2%) %
Sex				
Male	41.0*	27.3*	38.5*	27.0*
Female	59.0*	72.7*	61.5*	73.0*
Age Group				
Youth (18 - 25 years)	17.9	24.9	25.0	23.5
Adult (26 - 45 years)	64.1	52.4	54.8	54.0
Middle age (46 - 65 years)	17.9	22.7	20.2	22.5
Employment status:				
Employed	15.4	12.3	9.6	13.6
Self-employed	74.4	72.3	72.1	72.7
Unemployed	10.3	15.4	18.3	13.6

* *P*< 0.05

WHO (2011) recommends 300 minutes per week of moderate-intensity physical activity and 150 minutes per week of vigorous-intensity physical activity for an individual between the ages of 18 years to 64 years old. The mean moderate-intensity physical activity time by respondents was 122 minutes/week (min/wk), which is lower than the recommended 300 min/wk and 236 min/wk of vigorous-intensity physical activity which met the recommended 150 min/wk. There were 87% of physically low active respondents (< 150–300 min/wk).

Male respondents engaged more in moderate and vigorous-intensity physical activity at an average time of 281 min/wk and 309 min/wk respectively; this was significantly different from the female. Youth engaged more in both moderate and vigorous-intensity physical activity at 227 min/wk and 208 min/wk respectively, significantly more than the combined average time recorded by an adult (97 min/wk & 82 min/wk) and middle age (69 min/wk & 54 min/wk) respondents. There was no significant difference in the moderate- and vigorous-intensity physical activities across respondents' education. Respondents earning more than 20,000 naira monthly had a significantly higher moderate-intensity and vigorous-intensity physical activity duration at 144 min/wk and 132 min/wk, although they did not meet either of the WHO recommendations. Physical activity duration was not significantly different for moderate-intensity and vigorous-intensity physical activities across respondents' years of residence, employment status, and household size (Table 4).

**Table 4:** Physical activity duration according to demographic characteristics

Variables	Moderate-intensity physical activity (122 ± 325.35) min/week	Vigorous-intensity physical activity (236 ± 742.34) min/week
Sex		
Male	281^a^	309^a^
Female	56^b^	21^b^
Age Group		
Youth (18 - 25 years)	227^a^	208^a^
Adult (26 - 45 years)	97^b^	82^b^
Middle age (46 - 65 years)	69^b^	54^b^
Education		
No formal education	109	121
Primary school education	105	117
Secondary school education	118	117
College/University education	77	138
Years of residence		
10 years or less	125	111
11 years to 20 years	93	111
21 years to 30 years	160	105
More than 30 years	93	46
Employment status		
Employed	118	160
Self-employed	118	124
Unemployed	35	81
Monthly income		
No income	25^b^	19^b^
20,000 naira or less	124^ab^	105^ab^
More than 20,000 naira	144^a^	132^a^
Household size		
1 member	149	159
2 to 4 members	120	105
5 or more members	119	96

*Alphabet indicator shows significant difference

**Fig 1:**
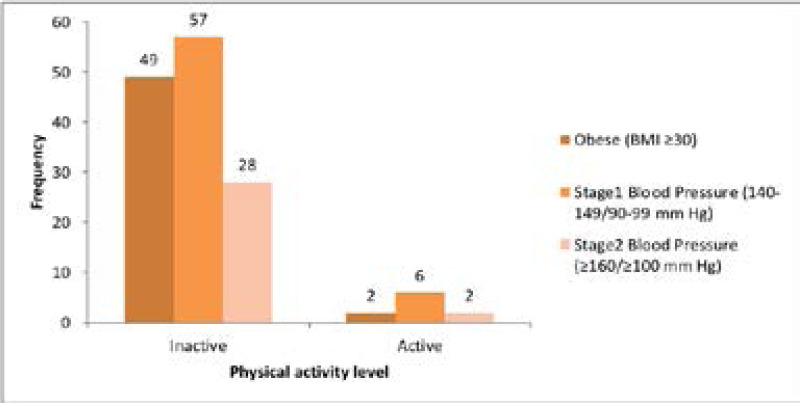
respondents' physical activity level with blood pressure level and obesity

The categorisation of respondents according to their physical activity level with blood pressure and BMI-categorised obesity is shown in Figure 1. Physically low active respondents accounted for the most proportion of obese individuals (96.1%), stage one blood pressure individuals (90.5%), and stage two blood pressure individuals (93.3%).

## Discussion

The purpose of this study was to estimate the level of physical activity and dietary patterns of residents in urban settlements in Ibadan. The prevalence of low physical activity at 87.2% reported in this study is higher than that reported by Hallal^20^ at 27.5%. Equally, low physical activity over time has been associated with increased risk of chronic diseases^36^ and individuals at risk are the employed populace, especially white-collar jobs workers. Findings from this study highlighted a low prevalence of workplace moderate-intensity and vigorous-intensity physical activities, and lower among employed respondents. This group of individuals are characterised by prolonged sitting time at work, a behaviour linked to premature death even if regular physical activity is engaged in^37^.

In a Nigerian study, workplace physical activity was found to be lower among bankers^16^ and Akindutire et al.^38^ suggested that these group of individuals are more at risk of chronic diseases associated with low physical activity. In their study, the authors further proposed the need for gaining a better and well-grounded understanding of the dynamics of physical activity in workplaces^38^. Several studies have however acknowledged that physical activity in the workplace is influenced by the availability of sports facilities and equipment, time constraints, job satisfaction, and their awareness of physical activity benefits^39^,^40^. Findings show that physical activity level was found to be below the WHO recommendation of 300 minutes per week. However, youths spent significantly more time in moderate-intensity physical activity (227 min/wk) compared to the older adults. This is rightfully so as the age of 19 to 24 years has been suggested as the best period to inspire and develop regular physical activity behaviour^41^. Consequences of the low physical activity among the younger generation are mostly shown in the later stages of life^42^, during which non-communicable diseases sets in. This is creating a shift from communicable disease to more non-communicable disease burden in Nigeria^43^. Low physical activity behaviour is formed during adolescence into early adult age and deteriorate with age, which corroborates studies conducted by Bauman et al.^44^ and Sallis et al.^45^, and as shown that older respondents spent the lowest time engaging in physical activity.

Fruits and vegetables have been estimated to potentially save up to 2.7 million lives if they are sufficiently consumed^46^; this is attributable to the low glycaemic index which has been associated with low risk for CVDs^47^. However, regular consumption of fruits and vegetables was low among respondents. According to Ogundari et al.^48^, income plays a significantly influential role in the consumption of fruits as wealthier households tend to consume more. This was however not in conformity with findings from this study; households earning less than the national minimum wage tend to consume fruits more than the wealthier households. While Powel et al.^49^ argued that fruits and vegetables are often not simply available to low-income earners due to its high price, studies by Bondoin^50^ and Hodder^51^ indicated that high cost does not influence low consumption as with reduced cost, consumption was not significantly increased. A study by Bokeshemi et al.^52^, stated that consumption of fruits and vegetables was rather also dependent on their local and seasonal availability, social preference, cultural value, and their importance to people.

Healthy diet and exercise are important in the prevention of NCDs in populations. A combination of changes in diet and physical exercise will have resultant effects on Africans who are overweight or obese. An attempt at reducing an unhealthy diet in the absence of high physical activity may prove futile as these phenomena are not independent of each other in the control of cardiovascular diseases.

## Conclusions

This study has brought to light that the prevalence of low physical activity is high among respondents, males were more physically active than females. Findings also show low intake of vegetables and fruits among respondents. Study findings implies that the risk of developing CVDs is potentially higher among females compared to males. The younger respondents were expectedly more physically active than the older adults, a trend that indicates a reduced likelihood of healthy aging. Coupled with low physical activity, respondents' consumption of fruits and vegetables was low; this was significantly lower among employed respondents and with those with lower level of physical activity being at higher risk of the consequences. In conclusion, study findings highlight low physical activity levels in semi-urban areas and also poor dietary patterns as exhibited in the low consumption of fruits and vegetables. This highlights the need for public health interventions to promote healthy diet and active life style among semi-urban areas.

## Study Limitations

This study was cross-sectional and limited to individuals between the age of 18 years and 65 years old in semi-urban settings in a city in Nigeria. Thus, findings may not be generalisable to other settings, including rural communities. This study findings may not be generalisable to other geographical areas in Nigeria due to cultural and socioeconomic diversity. Also, study tools were based primarily on self-report which may give room for bias.

## Declarations

### Availability of data and materials

The dataset used and/or analysed during the current study are available from the corresponding author on reasonable request.
